# Intestinal Nematode Infection Confers a Benefit to a Non‐Declining Frog Species, While a Fungal Parasitic Infection Has Sublethal Impacts on Reproductive Investment

**DOI:** 10.1002/ece3.72053

**Published:** 2025-08-28

**Authors:** Danielle K. Wallace, Emma K. Bowman, Chloe Roberts, Elizabeth Hamshaw, Wanyue Ma, Lucas G. Huggins, Tanapan Sukee, Alexander S. Wendt, Laura A. Brannelly

**Affiliations:** ^1^ The Melbourne Veterinary School The University of Melbourne Werribee Victoria Australia

**Keywords:** chytridiomycosis, coinfection, helminth, nematode, reproduction, sub‐lethal effects

## Abstract

Emerging infectious disease is a major cause of wildlife decline around the world. Understanding the impacts of disease even in non‐declining populations is important for understanding population‐level health and resilience to other emerging threats. In this study, we explored the sublethal impacts of the fungal pathogen *Batrachochytrium dendrobatidis*, *Bd*, and a novel intestinal nematode on the non‐declining Australian frog, *Litoria lesueuri*. We collected male animals that were either infected with the fungal parasite, infected with a nematode parasite, infected with both parasites, or uninfected and brought them into the lab and monitored their morphology and fungal pathogen load over a 7‐week trial. At the end of the experiment, we dissected the animals, collected the testes, and identified their nematode prevalence and burden. We morphologically and molecularly characterised the intestinal nematode as belonging to the genus *Parathelandros* within the order Oxyuridae. We found that this *Parathelandros* sp. infection was beneficial to the adult frogs, where infected animals were larger and had larger forearm width (an important trait for mating) when we accounted for animal size. The exact mechanisms of this improved condition are unknown and require additional research. However, in the *Bd*‐infected animals, we found an overall negative impact of infection, including reduced forearm width and sperm production. *Bd* infection is prevalent in this species, and there are tangible sublethal impacts of *Bd* infection, indicating that this species is affected even if mortality due to disease is low.

## Graphical abstract

Understanding the impacts of disease even in non‐declining populations is important for understanding population level health and resilience to other emerging threats. In this study we explored the impacts of co‐infection of the fungal pathogen and a novel nematode parasite on a common Australian frog species. We found that this novel nematode actually confers a benefit to the frogs, where they were larger and had larger forearms while the fungal parasite had overall a negative effect on the frogs.

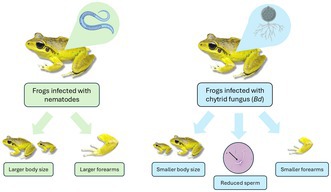

## 1 Introduction

Emerging infectious pathogens and parasites can have dramatic impacts on wildlife, but the impacts are often underreported. As such, the rates of decline and even extinctions in wildlife due to infection are likely underestimated in the literature (Preece et al. 2017). While disease can have dramatic effects on population health and stability in the long term, the effects are not always obvious. Species that have higher tolerance or resistance to infection can experience sublethal effects of infection (e.g., reduced body condition, locomotion, and changed behaviour; Bower et al. 2019; Brannelly, Chatfield, et al. 2018; Chatfield et al. 2013), which can have population‐level impacts on population size, longevity, and resilience (Boots and Norman 2000). Therefore, it is critical to investigate the impacts of disease on declining species to directly understand and address conservation and management concerns; however, it is also critical to investigate infection in non‐declining species. If these non‐declining species with infection are negatively impacted, their populations might be less resilient against other threats like climate change, human impacts, or another pathogen.

In addition to understanding the sublethal effects of disease on non‐declining species, it is important to identify how or if infection from one pathogen can influence the susceptibility and impact of another infection. Coinfections—simultaneous infection with multiple pathogens or pathogen strains—are common in wildlife, but the impacts are understudied (Hoarau et al. 2020). Coinfections and the pathogens' influence on disease outcomes (e.g., infection severity, survival, sublethal effects) are varied and often species (both the host and the pathogen species) specific and can have major health and population‐level implications that can take several forms (Bordes and Morand 2011). Coinfections can show (1) synergistic interactions, in which the presence of one infectious agent facilitates coinfection; (2) antagonistic interactions, in which the presence of one infectious agent inhibits coinfection; or (3) no interactions, where the presence of one infectious agent does not affect the activity of another (Bordes and Morand 2011; Hoarau et al. 2020). Understanding the impacts of coinfections, and how one pathogen influences another in wildlife as well as the sublethal effects of diseases can help us understand population‐level resilience and predict the risk of further declines. The sublethal effects of infection can reveal declining population trends in otherwise common species of ‘least concern’ that would have gone unnoticed.

One of the most dramatic and significant wildlife diseases in recent history is chytridiomycosis, caused by the fungal pathogen *Batrachochytrium dendrobatidis*, *Bd* (Skerratt et al. 2007). Part of the complexity of *Bd* infection dynamics is that some species can tolerate high infection loads or can clear infection, whereas others are highly susceptible to chytridiomycosis and succumb rapidly to infection (Brannelly, McCallum, et al. 2021). In species that are not declining, there are clear examples of how this infection causes sublethal effects. For example, infection can cause behavioral changes that can lead to higher rates of capture (Brannelly, Chatfield, et al. 2018), reduced locomotory function (Chatfield et al. 2013), and reduced metabolism and body condition (Campbell et al. 2019). Interestingly, there is evidence that frogs with active *Bd* infections will even increase their reproductive effort (Brannelly et al. 2025; Brannelly, Webb, et al. 2021; Brannelly et al. 2016; Chatfield et al. 2013), although there is also evidence that for some frog species, infection causes no change in reproductive effort (Greenspan et al. 2016) or will decrease reproductive effort (Brannelly, Webb, et al. 2021; Campbell et al. 2019). Understanding how infection affects reproduction is particularly important when investigating non‐declining species because changes in recruitment patterns and reproductive effort can impact population‐level stability and population size (Müller et al. 2019; Walton et al. 1997). Therefore, sublethal effects on breeding and reproductive effort can directly impact fitness of the individual, which can lead to population‐level changes.

The individual and population‐level implications of coinfection with *Bd* can vary across species because frogs exhibit different immune responses to *Bd* infection (Brannelly, McCallum, et al. 2021). Frogs tend to demonstrate a limited immune response to *Bd* infection (Voyles et al. 2009). Yet, despite the sheer volume of *Bd*‐related research, the interplay between *Bd* and other parasitic coinfections remains largely underexplored, much like other wildlife coinfections. The most studied coinfection with *Bd* to date is Ranavirus, but even then, the results are inconsistent across study locations and species (Ramsay and Rohr 2023; Warne et al. 2016; Whitfield et al. 2013). Nonetheless, the most common coinfection relationship is no interaction between the pathogens. Interestingly, infection with *Bd* or Ranavirus and subsequent coinfection with the other parasites can result in increased loads of both parasites (Ramsay and Rohr 2021).

There is relatively little known about the effects, distribution, and presence of macroparasites in amphibians. Nematodes are one type of helminth parasite that can impact amphibians. Amphibians are infected by a number of parasitic nematodes, and parasitic nematodes that infect the intestinal tract are likely common in wild frogs. However, nematode species are not commonly identified, and their direct impacts are understudied (Aho 1990; Santos et al. 2018). Here, we identified a novel nematode species and identified sublethal effects of infection on the amphibian, as well as the impacts of coinfection with *Bd*. This research helps to fill in critical research gaps about the impacts of these parasites on amphibian health.

In this study, we aimed to understand if infection and coinfection of *Bd* and nematode parasites are correlated with multiple aspects of morphology and male reproductive traits in the common and wide‐ranging stony creek frog (*Litoria lesueuri*). We collected naturally infected male animals from the field and brought them into the lab, where we maintained them individually and monitored aspects of body size and morphology (mass, length, forearm width) as well as *Bd* infection dynamics for 7 weeks. We analyzed testis morphology and spermatogenesis and identified the nematode infection status and load in the intestinal tract and lung of the animals at the end of the experiment. Intestinal tract nematodes were morphologically assessed and genetically sequenced to identify the nematode species. Through this study, we assessed the rate of coinfection and the impact that infection with these two parasites had on morphology and breeding effort.

The animals in this study were wild collected, and we allowed their natural infections to develop. It was not a randomized controlled trial but rather an observational study under controlled conditions. We do not know the previous exposure history of these animals to other parasites or pathogens and do not know the age of the animals. Therefore, there are limited causal inferences that we can conclude in this study. However, this study is an important first step in exploring the sublethal effects of parasites and pathogens on physiology and male reproduction in a non‐declining, stable species.

## 2 Methods

### 2.1 Field Collection and Husbandry

Male *L. lesueuri* (*n* = 32) were collected from the wild (see Appendix S1 for site information). Only males were used in this study because an aim of this study was to explore the impacts of infection on male reproductive traits. Therefore, the study implications are only relevant to males of this species. The animals were not treated for *Bd* infection on arrival in the lab, and any naturally acquired infection from the wild was allowed to develop during the experiment.

Frogs were housed in individual tanks (230 × 150 × 130 mm) on a gravel and moss substrate. Animals were maintained in the lab at an average temperature of 18°C, with a range between 15°C and 23°C. Enclosures were flushed daily with filtered water, and frogs were fed small crickets *ad libitum* twice per week. All animals were weighed (digital scale to the nearest 0.01 g), measured (snout‐to‐vent length, SVL, using dial callipers to the nearest 0.1 mm) and swabbed for *Bd* infection (see methodology below) in the 2 days of collection from the wild. Swab samples were taken weekly, and measurements were also taken weekly except for week six due to logistical issues. The width of the right forearm and the width of the nuptial pad were measured to the nearest 0.01 mm using digital callipers. We took three repeat readings per frog for the nuptial pad and arm width.

The experiment ended after 7 weeks, where all remaining animals were euthanized. Testes were removed, and the length of both testes was measured using digital calipers (0.01 mm) and then the gonads were placed in formalin for histological processing. The whole specimens were then placed in buffered formalin. For the sample size of each infection group, see Table 1.

**TABLE 1:** The *Bd* and nematode infection status at the end of the experimental timeframe: 7 weeks after the animals were taken into captivity. Nematode positive means that adult female nematodes were present in the intestinal tract of the animals at euthanasia, and Nematode negative means no adult female nematodes were found in the intestinal tract. *Bd* infected means they were infected at the time of euthanasia, *Bd* uninfected means the animals were never positive for *Bd* during the entire experiment, and *Bd* cleared means the animals were negative for *Bd* for at least 2 weeks before the end of the experiment.

	Nematode negative	Nematode positive	Totals
*Bd* uninfected	6	5	11
*Bd* infected	10	3	13
*Bd* cleared	2	6	8
Totals	18	14	Total: 32

### 2.2 Testing for Bd Infection

We standardized the swabbing procedure by performing five repeat swab strokes on the middle of the venter, side of the venter, each thigh, and limb of each frog. Samples were stored at −20°C until processing. The DNA from the skin swabs was extracted using a standard Prepman Ultra (Applied Biosystems, Life Technologies Pty Ltd) procedure (see Appendix S1). The DNA was diluted 6:100 and analyzed using standard qPCR (Rotogene, Qiagen) methodology (Boyle et al. 2004) with minor modifications (Appendix S1). We ran each sample in singlicate for 40 cycles (Roto‐Gene Q 2.3.5 software). In each qPCR reaction plate, we included a set of seven standards of known *Bd* concentrations (Pisces Molecular) and a no‐template control. We calculated infection load as *Bd* ITS DNA copies present on the whole swab (Appendix S1). We considered the sample to be positive if > 2 ITS DNA copies were present in the reaction well. Animals were considered infected if they had at least one positive swab sample throughout the experiment. Animals were considered cleared of infection if they had at least two consecutive negative swab results and remained negative to the end of the experiment.

### 2.3 Testes Preparation and Morphology

The formalin‐fixed left testis (*n* = 30, with two samples not included due to issues with sample preservation) was analyzed for testis morphology and spermatogenesis. Testes were dehydrated in a graded series of ethanol, cleared with xylene, and embedded in paraffin. Two sequential 5 μm transections of tissue were taken from six random locations (total of 12 histosections) along the length of the testis between the pole and center. The sections were mounted on glass slides, stained with haematoxylin and eosin stain, and cover slipped. Six non‐sequential histosections (one histosection randomly chosen from the pairs of sequential histosections) were examined for testis morphology for each animal. Photographs were taken of the whole histosection to calculate histosection area and count the number of seminiferous tubules at 40× magnification. In each histosection analyzed, the two largest seminiferous tubules were identified and photographed at 200× magnification, where tubule area was measured and both the maximum and minimum germinal epithelium depth were measured within each of the largest tubules (Appendix S1). The photos were analyzed using ImageJ (Schneider et al. 2012).

To assess spermatogenesis activity within the testis, we analyzed the number of cell clusters of each sperm stage within the testis. We chose four non‐sequential histosections, and within each of those four histosections, we identified the two largest seminiferous tubules. At 400× magnification, we took photos that included the germinal epithelium of each of those two largest seminiferous tubules. We then identified the number of cell clusters within that field of view that belonged to each of the four distinct stages of spermatogenesis: spermatogonia, spermatocytes, spermatids, and spermatozoa (Brannelly, Webb, et al. 2021; Brannelly et al. 2016).

### 2.4 Parasitic Nematode Collection

During dissection, nematode samples were taken from two male frogs, and these samples were preserved in 95% ethanol and stored at −20°C. Approximately 6 months after euthanasia (*n* = 32), dissection, and fixation in formalin, we dissected each frog to remove the lungs and intestine. The lungs were placed on a glass slide and observed under a light microscope (Leica LED2000) to examine for the presence of lungworm. No lungworm was found in any of the samples. We then removed the large intestine and analyzed the faeces for intestinal nematodes. We removed the faeces by making an incision at the distal end of the large intestine and applying pressure to compress the large intestine. We made one or two faecal smears depending on the volume of faeces removed from the frog. The faecal smears were observed under a light microscope for the presence of adult female nematodes (Appendix S1).

### 2.5 Morphological and Molecular Characterization of Nematodes

Identification of nematodes was made on the basis of morphological features. Nematodes were cleared in lactophenol, mounted on slides, and examined under a compound microscope.

Adult nematode samples fixed in ethanol at the time of euthanasia were thawed and washed with PBS to remove ethanol. Nematode DNA was extracted using the Qiagen DNeasy Blood and Tissue Kit as per the manufacturer's instructions (Qiagen, DEU). The only modifications to the extraction protocol were that a 1‐h proteinase K digestion step was used and that two elution steps were performed instead of one, the first with 40 μL and the second with 30 μL of elution buffer.

Six worm‐extracted DNA samples were then subjected to a nanopore‐sequencing based assay for the molecular characterisation of nematodes (Appendix S1). Nanopore sequencing was conducted on the MinION Mk1B platform (Oxford Nanopore Technologies, Oxford, UK), using the PCR Barcoding Expansion 1–96 (EXP‐PBC096) with Ligation Sequencing Kits (SQK‐LSK110). The laboratory and bioinformatic pipeline were carried out following Huggins, Atapattu, et al. (2024); Huggins, Colella, et al. (2024) (Appendix S1).

### 2.6 Phylogenetic Analysis

Parasitic nematode 18S rRNA gene sequences were downloaded from GenBank, imported into Geneious Prime 2024.0 (Biomatters, Auckland, New Zealand), and aligned using MUSCLE (Edgar 2004) alongside the dominant sequence obtained using nanopore sequencing in this study. The best nucleotide substitution model for the alignment was determined with maximum likelihood analysis, and the model with the lowest Bayesian Information Criterion (BIC) value was selected for phylogenetic inference. Neighbour‐joining method analyses were conducted using the Kimura two‐parameter model, and the phylogenetic tree was constructed in MEGA 11 (version 11.0.13) (Tamura et al. 2021) with bootstrap analyses using 5000 replicates.

For Bayesian phylogenetic inference (BI) FASTA alignments were converted to NEXUS format with BI trees inferred using MrBayes (version 3.2.7) (Ronquist et al. 2012). Each BI was performed with two million Markov Chain Monte Carlo (MCMC) generations, sampling every 100th generation with four chains by allowing for transitions and transversions with gamma‐distributed rates. Trees outputted by MrBayes were imported into FigTree (version 1.4.4.) and then Adobe Illustrator version 27.3.1 (Adobe, San Jose, USA) for editing and merging of bootstrap and posterior probability values, as well as to improve clarity.

### 2.7 Statistical Analysis

All statistical analyses were conducted in R using the R‐Studio interface (R Core Team 2022; RStudio Team 2020). For linear or generalized linear mixed effects models (LME/GLME) and linear models (LM), we conducted model comparisons using Akaike's Information Criterion (AIC) where appropriate to assess the model with the best fit for the data. We chose the model with the lowest AIC as the best model. Models within two AIC were considered equal, and we then chose the simplest model. The models always included *Bd* infection status (infected, cleared and uninfected), nematode presence (nematodes were detected/nematodes were not detected at dissection). For *Bd* infection status, animals that never returned a positive swab result were considered uninfected, animals that were infected at the end of the experiment were considered infected, and animals that were initially infected but returned at least 2 consecutive negative swab results and remained negative through the end of the experiment were considered cleared from *Bd* infection. Week was included if weekly measures were taken and not included if only one timepoint was assessed (e.g., testis morphology). The interactive effect of *Bd* infection and nematode presence was included if it improved model fit, and if it was not included in the final model, it is understood that there was no significant interaction of *Bd* infection and nematode presence on the response variable. Body condition or size (mass or SVL) was included if it improved model fit. Model assumptions were assessed to ensure that none were violated. Where appropriate, we conducted Tukey's post hoc comparisons and determined effect size using Cohen's *d* statistic. Model details are provided in Appendix S1, and all model results are presented in Appendix S2, Table S1.

## 3 Results

### 3.1 Infection Dynamics

**FIGURE 1:**
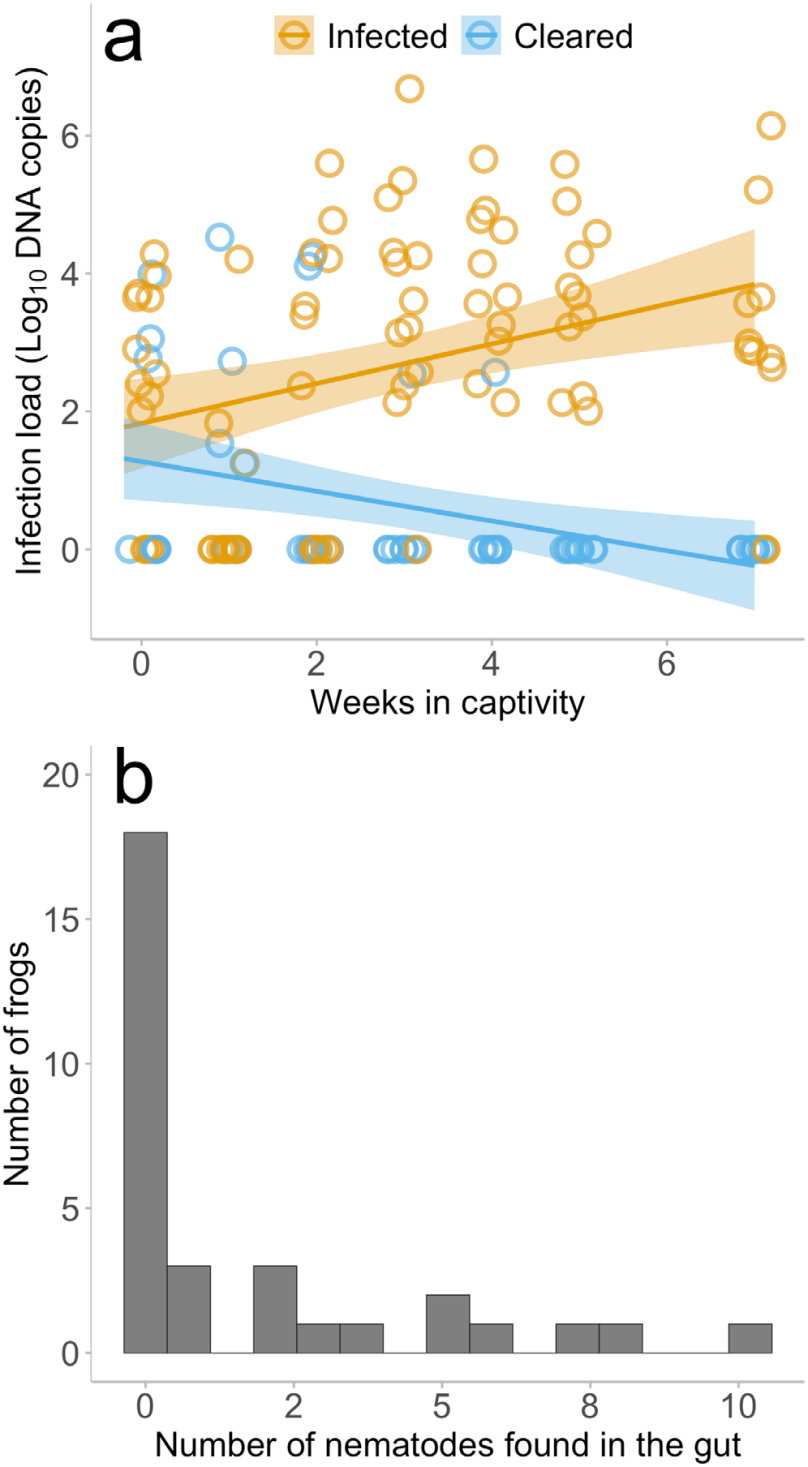
Infection dynamics and load of *Bd* infection and nematode infection. (a) The infection load of animals that returned at least one *Bd* + swab sample throughout the captive period (infected: *n* = 13; cleared; *n* = 8; in total: *n* = 21). Each point indicates one swab sample. Week 0 indicates the swab results from the day they arrived in captivity. The lines indicate the model and the shading around the line indicates smoothed conditional means. (b) The distribution of nematode load found within the samples. This figure indicates the 18 individuals that had 0 nematodes in their intestinal tract, and the 14 individuals that had between 1 and 10 nematodes.

Of the 32 animals that were used in this analysis, 65.6% of the frogs were infected with *Bd* (*n* = 21) upon arrival to the lab. Over the experimental timeframe, 38.1% (*n* = 8) (Table 1) individuals cleared their *Bd* infection. There was a significant interaction of week and *Bd* infection status on *Bd* infection load in animals that tested positive for *Bd* at least once throughout the 7‐week experimental timeframe (LME week × *Bd* infection load $\chi_{1}^{2}$ = 19.325, *p* < 0.001, Table S1). The animals that maintained *Bd* infection over the experiment had increasing infection load over time, while animals that cleared their infection had a decreasing load, where cleared individuals decreased load over the captive period (Figure 1a).

**FIGURE 2:**
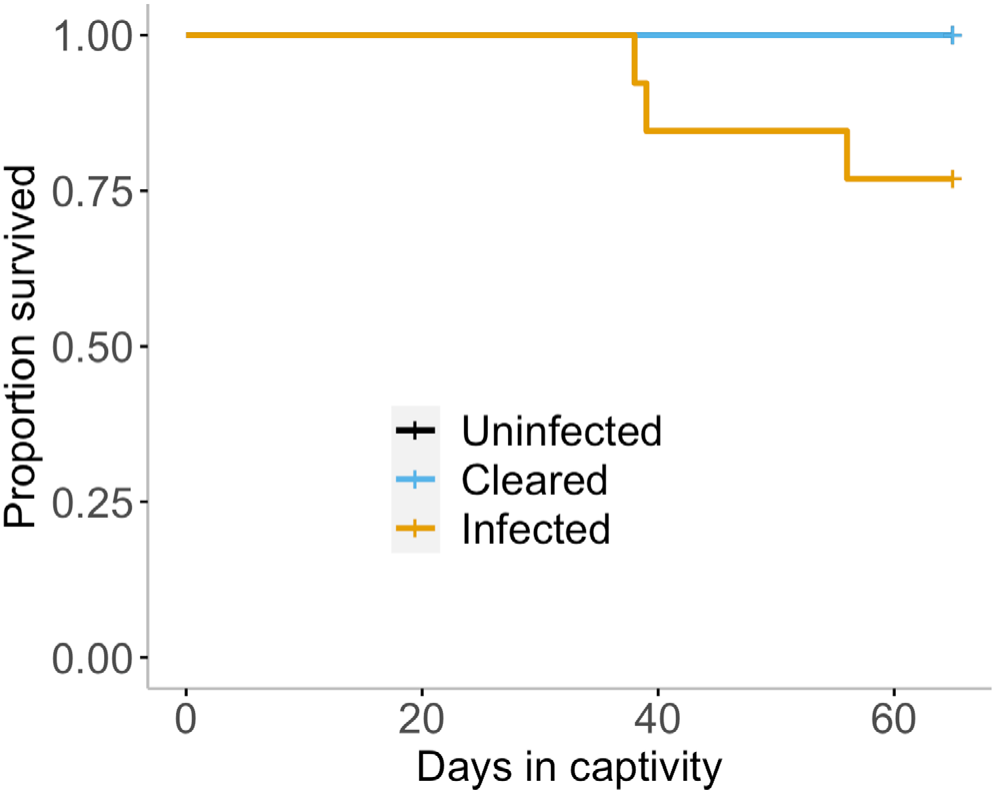
A survival curve over the 7‐week (65 day) experiment shown as animals infected with *Bd*: (uninfected: *n* = 11; infected: *n* = 13; cleared; *n* = 8). The lines represent proportion that survived over days of the experiment. When an animal displayed clinical signs of chytridiomycosis and lost its ability to right, it was humanely euthanised. Three animals were euthanised during the experimental timeframe. No animals that were uninfected with *Bd* needed to be euthanised, and no animals that cleared *Bd* infection needed to be euthanised. One animal that succumbed to chytridiomycosis was infected with nematodes (Day 39), and two were not (Day 38 and 56).

Three *Bd* infected animals (14.3%, of the 21 that were ever infected during the experimental period) succumbed to chytridiomycosis during this experiment and needed to be euthanized before the end of the 7 weeks (Day 38, 39, and 56). One of these animals had a nematode infection, and the other two did not. Even though it is clear that this species can succumb to chytridiomycosis, our survival analyses showed that there was no significant impact of chytrid infection on mortality within this experimental timeframe (Cox regression $\chi_{2}^{2}$ = 5.706, *p* = 0.058; Table S1; Figure 2).

Nematodes were found in 43.8% (*n* = 14 of 32) of the animal's intestinal tracts (Table 1). The number of adult female nematodes found in the intestinal tract ranged from 1 (3 individuals) to 10 adult worms (1 individual) (Figure 1b). Nematode eggs were not investigated.

In this study, we found no correlation between *Bd* infection status and the presence of intestinal nematodes in *L. lesueuri* (Pearson's chi‐squared test: $\chi_{2}^{2}$ = 5.445, *p* = 0.066).

### 3.2 Morphological Identification of Nematodes

The nematodes encountered in the intestine were considered to represent a species of *Parathelandros* Baylis, 1930, within the Oxyuridae (pinworm) family (Petter and Quentin 2009). Currently, seven species of this genus are known from Australian frogs (Barton 1994; Inglis 1968a, 1968b). The position of the excretory pore and the vulva at the level of the oesophageal bulb differentiates the current specimens from all congeners apart from *P. australiensis* (Johnston and Simpson 1942) and *P. lymnodynastes* (Johnston and Mawson 1949) which are both found in *Limnodynastes dumerilii* (Barton 1994). See Appendix S1 for female nematode measurement comparison and analysis.

Given the limitations of the available metric data, the specimens reported here are most similar to *P. australiensis* , based primarily on the length of the tail (Appendix S1 and Figure S1). In the absence of males, the precise species status of these specimens cannot be determined. No species of this nematode genus has been reported previously from *L. lesueuri* (Barton 1994), but part of its geographical range overlaps that of *Limnodynastes dumerilii*, the host of *P. australiensis* .

### 3.3 Molecular and Phylogenetic Characterisation of Nematodes

The dominant sequence from all six samples, when compared using blastn to the full NCBI GenBank database, was most similar to *Gyrinicola batrachiensis* (Accession: PP354087.1) with a 96.41% identity and 99% query cover. The next best matches were to the closely related *Gyrinicola gulabrevioris* (Accession: PP354091.1) and *Gyrinicola moohsia* (Accession: PP354088.1) with our sequence obtaining a 96.28% identity and 99% query cover match to both species. Given the relatively low percentage identity to any GenBank reference sequence, the nematode detected in the present study is likely a novel species within the genus *Gyrinicola* or represents a taxon from a molecularly uncharacterized but closely related genus within the Oxyuroidea (pinworm) family.

**FIGURE 3:**
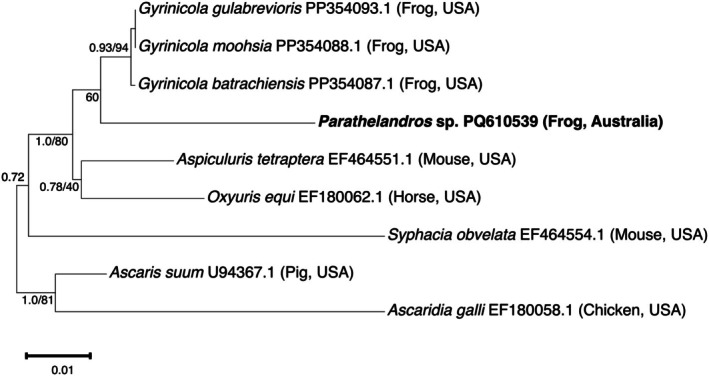
Phylogenetic relationship of the *Parathelandros* nematode (bold; NCBI accession number PQ610539) isolated from the anuran *Litoria lesueuri* alongside representative sequences from across the Oxyuridae. Phylogenetic inference was made using a neighbour‐joining distance and Bayesian method for a 782 bp segment of the 18S ribosomal RNA gene. Posterior probability values and bootstrap support (where available) for tree branches are indicated, with *Ascaris suum* and *Ascaridia galli* used as an outgroup. The scale bar length shows genetic difference as the number of nucleotide substitutions per site.

Next, the *Parathelandros* sp. sequence obtained in the present study was included within a phylogenetic analysis of a 782 bp region of the 18S rRNA gene from oxyurids and other nematodes (Figure 3). This analysis showed that the *Parathelandros* nematodes herein identified belong to a divergent but closely related genus to *Gyrinicola*, with both genera belonging to a single well‐supported clade (posterior probability of 1.0 and bootstrap support of 80%) that is separate from the mammal‐infecting oxyurids.

### 3.4 Frog Morphology

**FIGURE 4:**
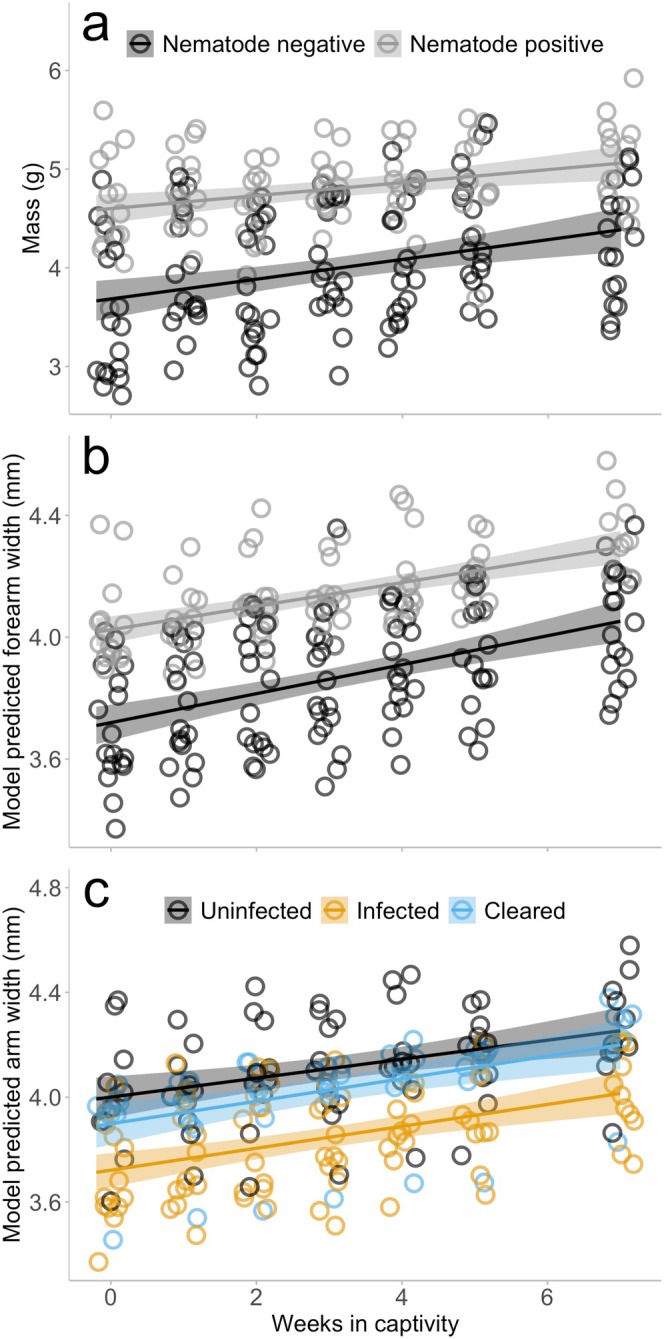
Morphology data, body mass (g) (a), and arm width (model predicted, mm) (b and c) recorded at weekly intervals after time of collection: (a) presence or absence of intestinal nematode parasite and body condition over the 7‐week experimental period. There was no effect of *Bd* infection on body size therefore there is no corresponding figure; (b) presence or absence of intestinal nematode parasite and model predicted arm width; (c) uninfected, infected, and cleared infection status for *Bd* and model predicted forearm width. Forearm width is presented as model predicted data because size of the animal is a significant covariate for forearm width; therefore, this presentation of the data accounts for the effect of size of the animal. There was no interactive effect of nematode and *Bd* infection on arm width, therefore we have presented the impacts of each parasite separately. The points represent individual weekly measurement taken (*n* = 32, measured weekly). The lines represent the model results, and the shading around the line represents smoothed conditional means.

Size of the animal (mass, g) was correlated with nematode presence and week, but not *Bd* infection (LME: nematode presence, $\chi_{1}^{2}$ = 14.804, *p* < 0.001; *Bd* infection, $\chi_{2}^{2}$ = 5.665, *p* = 0.059; week, $\chi_{1}^{2}$ = 87.846, *p* < 0.001; Table S1, Figure 4a). Size of all frogs increased over time, and the frogs with intestinal nematodes were 20.1% larger (4.80 ± 0.44 g) than frogs without gut nematodes detected (4.00 ± 0.66 g; *d* = −1.40).

Forearm width was influenced by *Bd* infection, nematode presence, week, and size (SVL) (LME: *Bd* infection, $\chi_{2}^{2}$ = 7.482, *p* = 0.024; nematode presence $\chi_{1}^{2}$ = 6.253, *p* = 0.012; week $\chi_{1}^{2}$ = 21.061, *p* < 0.001; SVL $\chi_{1}^{2}$ = 32.292, *p* < 0.001, Table S1). When we controlled for size (i.e., used model predicted results that included size as a covariate), animals that had nematodes in their intestinal tract had 7.4% larger forearms (4.15 ± 0.23 mm) compared to animals with no nematodes detected (3.87 ± 0.27 mm; *d* = −1.04; Figure 4c). In contrast, animals that remained infected with *Bd* throughout the experimental period had 6.6% smaller forearm width (3.85 ± 0.26 mm) than animals that were never infected with *Bd* (4.12 ± 0.29 mm; *d* = 0.99; Tukey's post hoc test, *p* = 0.057; Figure 4d). There was no significant difference in arm width between animals infected with *Bd* (Tukey's post hoc test; *p* = 0.096) and those that cleared infection, or between animals that cleared infection and those that remained uninfected (Tukey's post hoc test; *p* = 1.000).

There was no effect of either *Bd* infection or nematode infection on nuptial pad size or on scaled mass index (Table S1).

### 3.5 Testes Morphology Results

**FIGURE 5:**
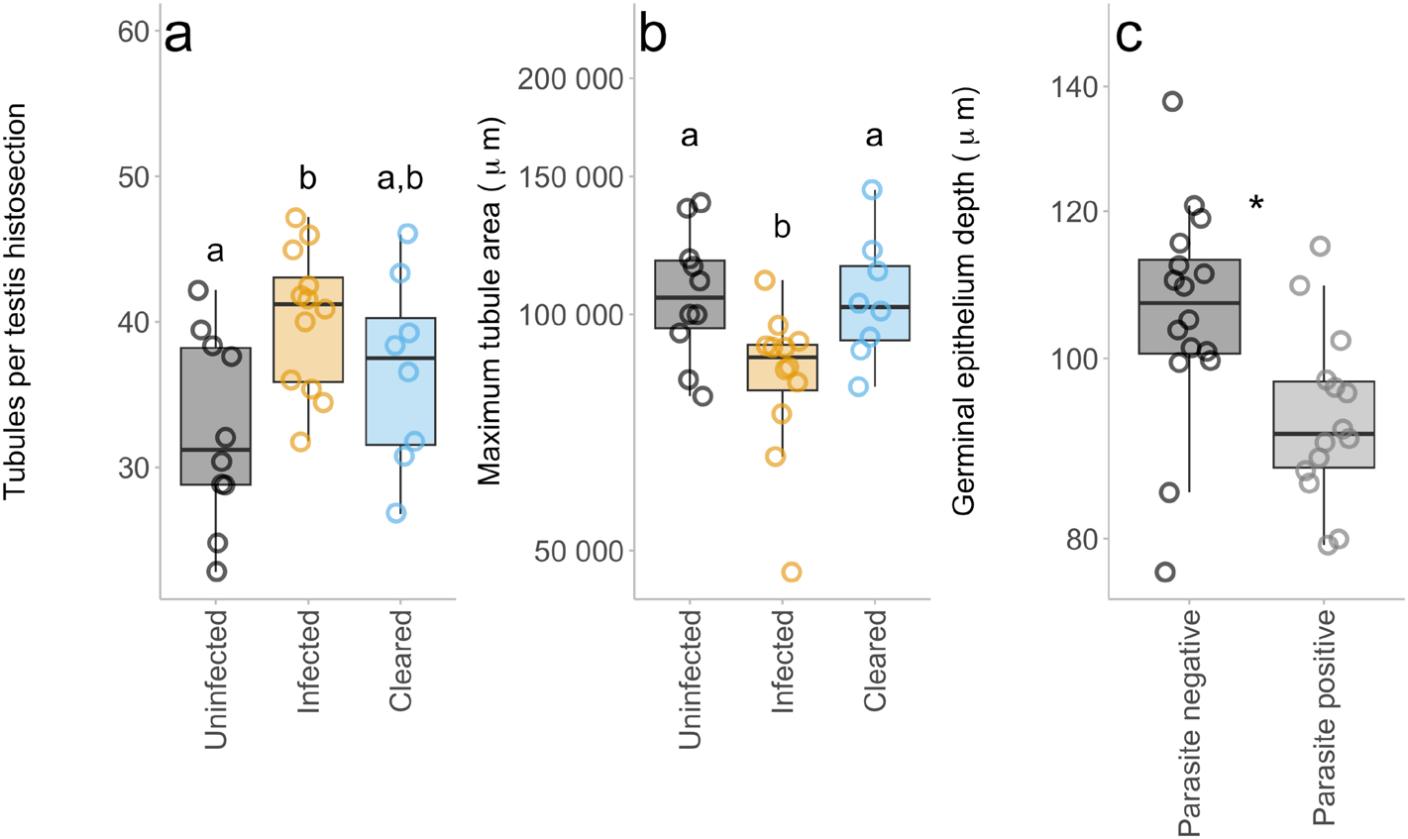
The impact of *Bd* infection or nematode presence on testis morphology: Tubules per testis histosection (a), maximum tubule area (b), and germinal epithelium depth. Within each panel (a and b) the “a” and “b” notation represents the results of the Tukey's post hoc tests, to indicate statistical difference amongst the groups. For panel c, * indicates a statistically significant difference between the two groups (parasite‐negative and parasite‐positive). The midline of the box plot represents the median value of the three samples taken per animal. Each point represents one individual (averaged values per each individual, *n* = 30), the upper and lower lines represent 1st and 3rd quartiles, and the whiskers represent 1.5× interquartile range.

Tubule count per histosection was significantly affected by *Bd* infection status but not by nematode presence (LME: *Bd* infection status, $\chi_{2}^{2}$ = 7.3197, *p* = 0.026; Table S1, Figure 5a)*Bd*‐infected animals had 22.9% more tubules per histosection (40.2 ± 5.90 μm^2^/tubule) compared to *Bd*‐negative animals (32.5 ± 7.68 μm^2^/tubule; *d =* −1.1; Tukey's post hoc, *p* = 0.031). There was no statistical difference between cleared and *Bd*‐negative or cleared and *Bd*‐positive individuals (*p* > 0.386).

The maximum tubule area was significantly affected by *Bd* infection status (LM: $\chi_{2}^{2}$ = 10.917, *p* = 0.004; Table S1, Figure 5b). *Bd*‐positive animals had a 22.2% smaller maximum tubule area (81,002 ± 1.29 μm^2^/tubule) than *Bd*‐negative animals (104,066 ± 1.34 μm^2^/tubule; *d* = 0.91; Tukey's post hoc, *p* = 0.010). *Bd*‐positive animals had 21.5% smaller average tubules than animals that cleared infection (103,183 ± 1.26 μm^2^/tubule; *d* = 0.89; Tukey's post hoc, *p* = 0.016). There was no significant difference in maximum tubule area between *Bd*‐negative animals and those that cleared infection (Tukey's post hoc, *p* = 0.980).

The maximum germinal epithelium depth of tubules was significantly affected by nematode presence but not *Bd* infection (LM: parasite presence, $\chi_{1}^{2}$ = 8.024, *p* = 0. Table S1; Figure 5c). Individuals with nematodes present had an 11.0% shallower germinal epithelium depth (91.3 ± 1.25 μm) than individuals without nematode parasites (103 ± 1.32 μm; *d* = 0.5). There was no statistically significant effect of *Bd* infection status or parasite infection on testis length (mm) or testis cross section area (Table S1).

### 3.6 Spermatogenesis

**FIGURE 6:**
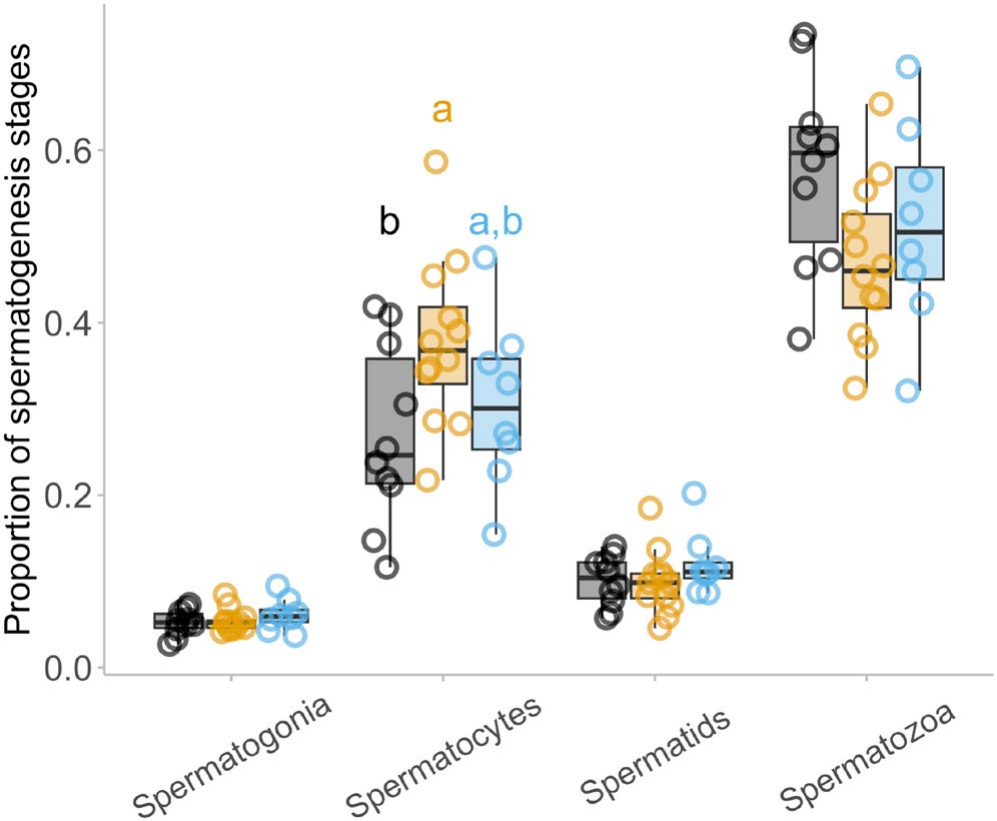
The proportion of spermatogenesis cell clusters within each spermatogenesis stage, as visualised across *Bd* infection statuses. The “a” and “b” notation above the Spermatocyte cell stage represents the results of the Tukey's post‐hoc tests, to indicate statistical difference amongst the groups. There were no statistically significant differences across the other spermatogenesis stages. The midline of the box plot represents the median value of the three samples taken per animal. Each point represents one individual (averaged values per individual, *n* = 30), the upper and lower lines represent 1st and 3rd quartiles, and the whiskers represent 1.5× interquartile range.

We found a significant effect of *Bd* infection but not parasite status on the proportion of spermatocytes present in *L. lesueuri* (GLME: Infection status, $\chi_{2}^{2}$ = 6.415, *p* = 0.040; Table S1, Figure 6). *Bd* infected animals had 25.0% more spermatocytes (median = 26.1%, IQR = 17.2%) than uninfected individuals (median = 34.8%, IQR = 18.6%; Tukey's post hoc test, *p* = 0.031; all other comparisons *p* > 0.376). There was no significant effect of *Bd* infection or nematode parasites on the proportion of spermatogonia, spermatozoa, or the total number of sperm cell clusters present in *L. lesueuri*.

## 4 Discussion

In this study we found no correlation in the coinfection of the *Bd* fungal pathogen and presence of intestinal nematodes in *L. lesueuri*. Interestingly, the identified *Parathelandros* nematode appears to have an overall positive effect on this frog through both animal size and forearm width. However, *Bd* infection had an overall negative impact on these frogs via smaller forearm width and testis morphology associated with reduced spermatogenesis. In infected animals that can fight infection, resources are often allocated to mounting a costly immunological response (Gadgil and Bossert 1970; Peterson et al. 2013); therefore, the sublethal impacts of *Bd* are expected in this species. In contrast, the observed physiological benefits of nematode infection are surprising, and such benefits are rarely reported in amphibians (but see Comas et al. 2014). Much of the literature on frogs and intestinal nematode parasites indicates that these parasites have negative sublethal impacts on frogs (Hausfater et al. 1990; Madelaire et al. 2013; Ramsay and Rohr 2023, 2021).

Our findings show that *Parathelandros* nematode infection was correlated with increased mass in *L. lesueuri*. Because this study collected animals from the wild with active infections and we did not expose the individuals to infection, we can only report that there is a correlation between parasite presence and size. Perhaps, bigger individuals were more likely to gain parasitic infections. Higher parasitic load can be correlated with higher body weight and/or size (Comas et al. 2014; Kelehear and Jones 2010). Larger size might increase the risk of transmission if the infection is transmitted orally, because larger‐bodied animals eat more and thus can increase their exposure to the parasite (Kelehear and Jones 2010). However, the larger bodies of the nematode‐infected frogs could be due to a release of chemicals within the intestine by the nematode such as hormone growth factors. Some nematodes such as the closely related *Gyrinicola* and certain cosmocercoid nematodes can release growth factors, prostanoids, or other chemicals that enable the nematode to create optimal host conditions (Pryor and Bjorndal 2005; Shu et al. 2022), and as a byproduct can cause the host to grow or increase feeding behavior. While these effects are documented in specific host–parasite systems, it is unclear whether similar mechanisms occur in *Parathelandros*, for which no such interactions have been described. Regardless of whether the increased size of nematode‐infected frogs is a cause or effect, it is well accepted that larger frogs are at a physiological advantage in the wild. For example, larger frogs tend to reach sexual maturity faster and produce more offspring (Gibbons and McCarthy 1986); they can eat larger prey and a larger variety of prey items (Lima and Moreira 1993), and can be less susceptible to infections like *Bd* (Brannelly, Martin, et al. 2018).

The animals infected with *Parathelandros* nematodes had larger forearms, even when we controlled for size. This result is unexpected because infections with parasites typically have negative impacts on the host (Hausfater et al. 1990; Madelaire et al. 2013; Ramsay and Rohr 2023, 2021). Forearm widths represent the size of the flexor carpi radialis muscles, which males use during mating (amplexus) to grip the female and coax her to release eggs in spawning (Greene and Funk 2009; Navas and James 2007; Oka et al. 1984). Forearm size is considered a secondary sexual characteristic in frogs because it is a trait that is sexually dimorphic, and a larger, stronger forearm can hold onto a potential mate for longer, hold a stronger grip, or squeeze more eggs during a spawning event (Greene and Funk 2009; Navas and James 2007; Oka et al. 1984). The finding that a *Parathelandros* sp. infection is associated with larger forearms in *L. lesueuri* male frogs indicates that infected individuals have a breeding advantage. While it is unclear if these nematodes caused the increase in arm size relative to body size, or if more robust animals that have more desirable secondary sexual characteristics (Greene and Funk 2009) are more likely to be infected, there is an association between robust animals and *Parathelandros* nematode infection.

We did find one negative effect of *Parathelandros* sp. infection on male *L. lesueuri* males: shallower germinal epithelium depth. Germinal epithelium depth is the tissue layer within the seminiferous tubules where the early stages of gametogenesis occur, including spermatogonia, spermatocytes, and spermatids (McCallum and Trauth 2007). A large germinal epithelium depth could mean more spermatogenesis activity, or it could mean a lower proportion of spermatozoa within the tubules. Furthermore, germinal epithelium depth in amphibian testes is highly variable across the testis, amongst the seminiferous tubules, and even within a single tubule (Brannelly, Webb, et al. 2021; Brannelly et al. 2016; McCallum and Trauth 2007). For this reason, the impact of germinal epithelium depth should be paired with other changes within the testis, like increased tubule size, or more spermatocyte activity. While our results might indicate that frogs with nematode infection are decreasing their effort in spermatogenesis, there are no other testis morphological changes associated with this, and thus this finding should be interpreted with caution.

Due to an absence of publicly available molecular data for the genus *Parathelandros*, the nematode that we identified within our study was molecularly most closely related to parasitic nematodes of the *Gyrinicola* genus. Both the *Parathelandros* and *Gyrinicola* genera are known to contain nematode species that infect amphibians (Inglis 1968a, 1968b; Walker et al. 2024). The 18S rRNA gene sequences from our *Parathelandros* nematode represent the first publicly available molecular data for this genus, due to a paucity of molecular analyses of Australian pinworms. Through phylogenetic analyses and comparison to GenBank sequences, it was shown to be highly divergent from any other publicly available reference sequences.

Oxyurid nematodes use a direct oral route of infection and complete their lifecycle in the aquatic environment. The prevalence of oxyurid nematode infections has even been documented in *ex situ* contexts, such as zoos (Huggins et al. 2017). There is little research into the health impacts of *Parathelandros* sp. in frogs, but a phylogenetically close and well‐studied oxyurid relative of amphibian‐infecting *Parathelandros* sp. is *Gyrinicola batrachiensis*, which appears to have developed a mutualism with herbivorous tadpoles. *Gyrinicola batrachiensis* has been found infecting several amphibian tadpole species across North America (Pryor and Greiner 2004), where infection leads to increased fermentation in the hindguts, resulting in higher energetic contributions, growth rates, and development times compared to uninfected tadpoles (Pryor and Bjorndal 2005). In this study, the improved physical condition of the *Parathelandros* sp. infected animals that we observed could be occurring through a similar mechanism to *G. batrachiensis* infection in other frog hosts. However, more research is needed to elucidate such mechanisms and obtain a better understanding of how this specific parasite influences its host.

Our study found that *Bd* infection had several clear sublethal effects on the *L. lesueuri* male frogs. Infected animals have smaller forearm width and reduced sperm production as evidenced by several testis morphology traits, including reduced seminiferous tubules area (which is the area in which sperm are produced inside the testis). We found an increase in the number of early stages of spermatogenesis (spermatogonia) in animals that were infected with *Bd*, but no change in the number of later stage spermatogenesis or total number of spermatogenesis cell bundles in the testes. Taken together, these findings indicate reduced reproductive effort in *Bd*‐infected male *L. lesueuri*. The reduced effort seen in *L. lesueuri* differs from other species that have shown increased effort in reproduction when infected with *Bd* (Chatfield et al. 2013; Brannelly et al. 2016; Brannelly, McCallum, et al. 2021; Brannelly, Webb, et al. 2021; Brannelly et al. 2025). This unusual finding highlights two key points. First, there is variability in how amphibian species respond to this devastating wildlife disease, such that understanding the impacts of infection is species and context dependent. Second, we observed low impact of infection on mortality in this study, and a high proportion of the animals cleared the infection, which might mean that *L. lesueuri* responds to *Bd* infection by decreasing investment in reproduction to reallocate resources to mounting a costly immunological response (Gadgil and Bossert 1970).

The similarity in reproductive effort between *Bd*‐cleared and uninfected *L. lesueuri* suggests that for those individuals able to clear infection, they can rapidly recover from the negative physical impacts of *Bd* infection. This finding differs from evidence in *Bd*‐infected male *L. aurea* , whose testes were reduced in size 5 months after clearing infection, likely indicating a slow recovery from infection in that species (Campbell et al. 2019). Importantly, *L. aurea* is highly susceptible to chytridiomycosis and suffers high mortality and rarely clears infection without environmental factors reducing *Bd* virulence (Campbell et al. 2019; Garnham et al. 2022). As such, disease dynamics within *L. aurea* are quite different from *L. lesueuri*. While there are clear negative impacts of disease on *L. lesueuri*, it does appear to recover quickly from disease.

*Litoria lesueuri* has been suggested to be a reservoir host for *Bd* in Victoria, specifically for the endangered frog species *L. spenceri* (West et al. 2024, 2020). We found that in the wild near metropolitan Melbourne, *L. lesueuri* has moderate prevalence of *Bd* infection that it can sustain for a long timeframe with low rates of mortality due to chytridiomycosis, such that there was no statistical difference in survival between animals that were infected and animals that never had infection. Maintenance of infection, minimal sublethal effects, and low mortality are important traits for competent reservoir hosts, like *Crinia signifera* and *Pseudacris regilla* (Brannelly, Webb, et al. 2018; Messersmith et al. 2024; Reeder et al. 2012). However, *L. lesueuri* has sublethal impacts of infection, and it also has high rates of *Bd* clearance, which indicates that it is not a competent reservoir host of infection, like other known amphibian reservoir hosts (Brannelly, Webb, et al. 2018; Reeder et al. 2012). There is conflicting information in the literature about *L. lesueuri* as a competent reservoir host, with some suggesting high mortality rates due to *Bd* (West et al. 2020) and others suggesting very low prevalence in this species (Crawford‐Ash and Rowley 2021; Rowley and Alford 2007). Our study along with previous literature indicates that in some locations, such as Victoria, *L. lesueuri* can act as a source of infection for sympatric declining species, but is likely not a competent reservoir host of *Bd* infection.

## 5 Conclusion

In this study we found two infectious agents that infect the non‐declining *L. lesueuri*: *Bd* fungal pathogen and a *Parathelandros* nematode species. The intestinal oxyurid nematode, *Parathelandros* sp. infection appears to have an overall positive impact on this species, and more work needs to be done to understand the causal relationship between nematode presence, size of the animal, and relative forearm size. It is possible that infection with *Parathelandros* sp. could act as a buffer or improve resilience within a population. In contrast, *Bd* infection appears to have an overall negative impact on this frog species. The sublethal effects of *Bd* infection affect several measures of reproductive fecundity, such that overall population resilience of the population is likely impacted when there is high disease prevalence. However, this species can clear *Bd* infection and return to baseline reproductive effort within several weeks, indicating that recovery from infection is quick.

Understanding sublethal effects of infection in non‐declining species is essential to predict how additional stressors might compromise population resilience. In a changing world with many different emerging threats to our ecosystem and wildlife, understanding the sublethal effects of one threat (e.g., infection) can help us predict the cumulative impacts of additional threats that might impact the system. For example, if disease is present in a stable non‐declining species but has sublethal effects on individuals, then population‐level resilience and stability might be threatened when fire, habitat destruction, or another disease enters the system.

## Author Contributions

**Danielle K. Wallace:** conceptualization (equal), investigation (equal), writing – original draft (equal). **Emma K. Bowman:** investigation (equal), writing – review and editing (equal). **Chloe Roberts:** investigation (equal), writing – review and editing (equal). **Elizabeth Hamshaw:** investigation (equal), writing – review and editing (equal). **Wanyue Ma:** investigation (equal), writing – review and editing (equal). **Lucas G. Huggins:** formal analysis (equal), investigation (equal), writing – review and editing (equal). **Tanapan Sukee:** formal analysis (equal), investigation (equal), writing – review and editing (equal). **Alexander S. Wendt:** investigation (equal), writing – review and editing (equal). **Laura A. Brannelly:** conceptualization (equal), data curation (equal), formal analysis (equal), funding acquisition (lead), investigation (equal), methodology (equal), project administration (lead), supervision (lead), writing – original draft (equal).

## Ethics Statement

The project was undertaken following University of Melbourne Animal Ethics application 10267, and Victoria Department of Environment, Land, Water and Planning Wildlife Act 1975 Research Authorisation permit number 10010313.

## Conflicts of Interest

The authors declare no conflicts of interest.

## Supporting information


**Appendix S1:** ece372053‐sup‐0001‐AppendixS1.docx.

**Appendix S2:** ece372053‐sup‐0002‐AppendixS2.docx.

## Data Availability

Data is provided through Data Dryad: Doi: https://doi.org/10.5061/dryad.f1vhhmh6c.
