# Supplementary material for: Intestinal Nematode Infection Confers a Benefit to a Non‐Declining Frog Species, While a Fungal Parasitic Infection Has Sublethal Impacts on Reproductive Investment
Source: Ecol Evol. 2025 Aug 28;15(9):e72053. doi: 10.1002/ece3.72053 (PMC12394065; doi:10.1002/ece3.72053)
Supplement: Supplementary file 1 — Appendix S1: ece372053‐sup‐0001‐AppendixS1.docx. [file ECE3-15-e72053-s002.docx]

**Title**: Intestinal nematode infection confers a benefit to a non-declining frog species, while a fungal parasitic infection has sublethal impacts on reproductive investment.

**Authors**: Danielle K Wallace, Emma K Bowman, Chloe Roberts, Elizabeth Hamshaw, Wanyue Ma, Lucas G Huggins, Tanapan Sukee, Alexander S Wendt, Laura A Brannelly*

Appendix 1

Table S1 – Statistical model results

**Supplemental Table**

Table S1| The full statistical results for all disease dynamics, morphological, and gonadal results for male *Litoria lesueuri* that had nematodes present in their intestinal tract, or had *Bd* infection on their skin over the 7-week study. The models performed were cox regressions (for survival analyses), Pearson’s chi-squared test, to determine the impact of coinfection, or a series of Linear models (LM), Linear mixed effects models (LME) and generalised linear mixed effects models (GLME) with a beta distribution. Df is degrees of freedom, * indicates the p values is less than 0.05, ** indicates the p value is less than 0.01.

| **Category** | **Model response variable** | **Sample size for each treatment group** | **Statistical model used** | **Fixed effects** | **Random effects** | **Random effect model result** | **Test statistic** | **df** | **p value** |
| --- | --- | --- | --- | --- | --- | --- | --- | --- | --- |
| Coinfection | *Bd* infection status (uninfected, infected, cleared) vs nematode presence (positive, negative) | N = 32 | Pearson’s Chi-squared test |  |  |  | 𝜒^2^ = 5.445 | 2 | p = 0.066 |
| *Bd* infection load | *Bd* load (log_10_ DNA copies) in infected and cleared animals | N = 22 (9 cleared and 13 infected) | LME | *Bd* infection status (cleared, infected) |  |  | 𝜒^2^ = 35.791 | 1 | p < 0.001** |
|  |  |  |  | Week |  |  | 𝜒^2^ = 2.416 | 1 | p = 0.120 |
|  |  |  |  | *Bd* infection status × Week |  |  | 𝜒^2^ = 19.325 | 1 | p < 0.001** |
|  |  |  |  |  | Frog ID | Variance = 0.289 ± 0.538 |  |  |  |
| Survival | Survival status (survived or humanely euthanised) and days survived | N = 32 | Cox regression | *Bd* infection status |  |  | 𝜒^2^ = 5.706 | 2 | p = 0.058 |
| Morphology | Size (mass, g) | N = 32 | LME | *Bd* infection status |  |  | 𝜒^2^ = 1.600 | 2 | p = 0.450 |
|  |  |  |  | Nematode presence |  |  | 𝜒^2^ = 0.195 | 1 | p = 0.659 |
|  |  |  |  | Week |  |  | 𝜒^2^ = 0.025 | 1 | p = 0.8755 |
|  |  |  |  |  | Frog ID | Variance = 0.017 ± 0.129 |  |  |  |
|  | Scaled mass index | N = 32 | LME | *Bd* infection status |  |  | 𝜒^2^ = 5.665 | 2 | p = 0.059 |
|  |  |  |  | Nematode presence |  |  | 𝜒^2^ = 14.804 | 1 | p < 0.001** |
|  |  |  |  | Week |  |  | 𝜒^2^ = 87.846 | 1 | p < 0.001** |
|  |  |  |  |  | Frog ID | Variance = 0.218 ± 0.467 |  |  |  |
|  | Forearm width (mm) | N = 32 | LME | *Bd* infection status |  |  | 𝜒^2^ = 7.482 | 2 | p = 0.024* |
|  |  |  |  | Nematode presence |  |  | 𝜒^2^ = 6.253 | 1 | p = 0.012* |
|  |  |  |  | Week |  |  | 𝜒^2^ = 21.061 | 1 | p < 0.001** |
|  |  |  |  | SVL (mm) |  |  | 𝜒^2^ = 32.292 | 1 | p < 0.001** |
|  |  |  |  |  | Frog ID | Variance = 0.009 ± 0.096 |  |  |  |
|  | Nuptial pad width (mm) | N = 32 | LME | *Bd* infection status |  |  | 𝜒^2^ = 0.117 | 2 | p = 0.943 |
|  |  |  |  | Nematode presence |  |  | 𝜒^2^ = 3.383 | 1 | p = 0.066 |
|  |  |  |  | Week |  |  | 𝜒^2^ = 53.562 | 1 | p < 0.001** |
|  |  |  |  | SVL (mm) |  |  | 𝜒^2^ = 18.212 | 1 | p < 0.001** |
|  |  |  |  |  | Frog ID | Variance = 0.009 ± 0.096 |  |  |  |
| Testes morphology | Testis length | N = 30 | LM | Infection status |  |  | F = 0.484 | 2 | p = 0.623 |
|  |  |  |  | Parasite status |  |  | F = 0.495 | 1 | p = 0.489 |
|  |  |  |  | SVL |  |  | F = 2.10 | 1 | p = 0.162 |
|  | Testis area | N = 30 | LME | Infection status |  |  | 𝜒^2^ = 2.079 | 2 | p = 0.354 |
|  |  |  |  | Parasite status |  |  | 𝜒^2^ =0.0001 | 1 | p = 0.992 |
|  |  |  |  | Frog mass |  |  | 𝜒^2^ =1.384 | 1 | p = 0.240 |
|  |  |  |  | Infection status × parasite status |  |  | 𝜒^2^ =1.131 | 2 | p = 0.568 |
|  |  |  |  |  | Frog ID | Variance = 9.236e+10 ± 303902 |  |  |  |
|  | Tubule count | N = 30 | LME | Infection status |  |  | 𝜒^2^ = 7.320 | 2 | p = 0.026* |
|  |  |  |  | Parasite status |  |  | 𝜒^2^ = 0.308 | 1 | p = 0.579 |
|  |  |  |  | Frog mass |  |  | 𝜒^2^ = 0.243 | 1 | p = 0.622 |
|  |  |  |  | Infection status × parasite status |  |  | 𝜒^2^ = 1.257 | 2 | p = 0.533 |
|  |  |  |  |  | Frog ID | Variance = 33.15 ± 5.758 |  |  |  |
|  | Maximum tubule area (log_10_  µm^2^) | N = 30 | LME | Infection status |  |  | 𝜒^2^ = 13.096 | 2 | p = 0.001* |
|  |  |  |  | Parasite status |  |  | 𝜒^2^ = 1.843 | 1 | p = 0.175 |
|  |  |  |  |  | Frog ID | Variance = 0.007 ± 0.084 |  |  |  |
|  | Maximum germinal epithelium depth | N = 30 | LME | Infection status |  |  | 𝜒^2^ = 2.824 | 2 | p = 0.244 |
|  |  |  |  | Parasite status |  |  | 𝜒^2^ = 8.024 | 1 | p = 0.005* |
|  |  |  |  |  | Frog ID | Variance = 0.0009 ± 0.096 |  |  |  |
| Sperm stage | Proportion of spermatogonia | n = 30 | GLME | Infection status |  |  | 𝜒^2^ = 2.259 | 2 | p = 0.323 |
|  |  |  |  | Parasite status |  |  | 𝜒^2^ = 1.376 | 1 | p = 0.241 |
|  |  |  |  |  | Frog ID | Variance = 0.05399 ± 0.232 |  |  |  |
|  | Proportion of spermatocytes | N = 30 | GLME | Infection status |  |  | 𝜒^2^ = 6.416 | 2 | p = 0.040* |
|  |  |  |  | Parasite status |  |  | 𝜒^2^ = 0.125 | 1 | p = 0.724 |
|  |  |  |  |  | Frog ID | Variance = 0.1868 ± 0.4322 |  |  |  |
|  | Proportion of spermatozoa | N = 30 | GLME | Infection status |  |  | 𝜒^2^ = 5.723 | 2 | p = 0.057 |
|  |  |  |  | Parasite status |  |  | 𝜒^2^ = 0.196 | 1 | p = 0.658 |
|  |  |  |  |  | Frog ID | Variance = 0.1549 ± 0.3936 |  |  |  |
|  | Total number of sperm cell clusters | N = 30 | LME | Infection status |  |  | 𝜒^2^ = 0.637 | 2 | p = 0.727 |
|  |  |  |  | Parasite status |  |  | 𝜒^2^ = 0.380 | 1 | p = 0.538 |
|  |  |  |  |  | Frog ID | Variance = 0.03285 ± 0.1774 |  |  |  |
