# Supplementary material for: Intestinal Nematode Infection Confers a Benefit to a Non‐Declining Frog Species, While a Fungal Parasitic Infection Has Sublethal Impacts on Reproductive Investment
Source: Ecol Evol. 2025 Aug 28;15(9):e72053. doi: 10.1002/ece3.72053 (PMC12394065; doi:10.1002/ece3.72053)
Supplement: Supplementary file 2 — Appendix S2: ece372053‐sup‐0002‐AppendixS2.docx. [file ECE3-15-e72053-s001.docx]

**Title**: Intestinal nematode infection confers a benefit to a non-declining frog species, while a fungal parasitic infection has sublethal impacts on reproductive investment.

**Authors**: Danielle K Wallace, Emma K Bowman, Chloe Roberts, Elizabeth Hamshaw, Wanyue Ma, Lucas G Huggins, Tanapan Sukee, Alexander S Wendt, Laura A Brannelly

Appendix 2

Supplementary methodology

Supplementary results

Figure S1

**Supplementary methodology and results**

***Methods***

*Study species*

The stony creek frog, *Litoria lesueuri*, is a common frog species that occurs along the south-east coast of Australia (Donnellan and Mahony, 2004). Populations of this species are stable and have experienced no declines due to *Bd*, yet are known to have a range of prevalence of infection in the field (Rowley et al., 2007; West et al., 2020). Direct evidence of mortality is rare, but chytridiomycosis deaths have been previously documented in wild individuals (Berger et al., 2005). While mortality of this species due to *Bd* appears uncommon, their widespread infection in the field could have sublethal effects on *L. lesueuri* populations that have not been evaluated, which might lead to long term impacts on population resilience.

*Field collection and husbandry*

Male *L. lesueuri* (n = 32) were collected from the wild at two sites in Lerderderg State Park (O’Briens Crossing, elevation 453m, -37.496026, 144.360978; Mackenzies Flat, elevation 33 m, -37.615696, 144.424809) in the austral summer of December 2022. *Litoria lesueuri* is sexually dimorphic, and males were distinguished by their characteristic yellow breeding colouration and their smaller size. Once captured in the field, individuals were maintained individually for biosecurity to ensure pathogens were not spread.

During the experiment, any animals showing signs of moderate to severe chytridiomycosis (loss of righting reflex, severe erythema of groin and venter, excess skin slough) were euthanised with an overdose of MS222 (Tricaine methanesulfonate, Sigma-Aldrich), and decapitation was used as a secondary kill procedure.

*Testing for Bd infection*

The DNA from the skin swabs was extracted in 50 µL Prepman Ultra (Applied Biosystems®, Life Technologies Pty Ltd) and 30–40 mg of 0.5 mm silica beads (Biospec). We homogenised the samples (using a cell homogeniser) for 2 min at 1400 oscillations per sec, then incubated the samples at 95 °C to lyse the cells for 10 min and collected and diluted the supernatant 6:100 in ultra-pure water before directly analysing for pathogen presence and quantity using qPCR (Brannelly et al., 2020). The remaining extracted DNA was stored at -20 °C. With every extraction series performed one *B. dendrobatidis* positive control sample (zoospores from culture), and one negative control (swab only) was extracted.

The diluted DNA extract samples were analysed using standard qPCR (Rotogene, Qiagen) methodology (Boyle et al., 2004) to identify and quantify *Bd* DNA in the samples with minor modifications. We ran our reactions at 15 µL volume with lo-ROX 2 × mastermix (SensiFast, Bioline), including BSA to reduce PCR inhibition plus 5 µL of template DNA per reaction well. We ran each sample in singlicate for 40 cycles (Roto-Gene Q 2.3.5 software). In each qPCR reaction plate, we included a set of seven standards of known *Bd* concentrations (Pisces Molecular) made using plasmid DNA of the *Bd* ITS region to confirm the *Bd* infection status and load from the skin swab samples. On every qPCR reaction plate, we included a no-template control (5 µL of ultra-pure water to replace the template DNA).

In some cases, animals came in with a negative swab result, but over the weeks in the lab they returned positive swab samples (n = 8). We considered these individuals as positive for infection because they likely came into the experiment with a low/undetectable infection load that gradually grew over time in captivity. While qPCR as a diagnostic tool is sensitive, low infection loads are often missed (Brannelly et al., 2020; Hollanders and Royle, 2022). We believe these animals had a low infection when they entered the facility that grew rather than contamination across tanks. We do not believe that these positive frog results were due to contamination because our biosecurity protocols were rigorous and individuals were maintained in individual enclosures.

*Testes preparation and morphology*

There is a developing understanding of how reproductive effort is being influenced by chytridiomycosis in amphibians, evaluated via proxies for gametogenesis such as gonad size and morphology, as well as secondary sex characteristics including colour and calling behaviour. Testis size in amphibians is closely tied to reproductive success, with an increase in size typically corresponding to greater sperm production (Brannelly et al., 2021, 2016; McCallum and Trauth, 2007), where increases in seminiferous tubule size (or reduced tubule density) indicate greater reproductive output – potentially due to an increase in intra-tubule space accommodating more mature spermatozoa (Brannelly et al., 2021, 2016; McCallum and Trauth, 2007). Germinal epithelium depth of seminiferous tubules has been used to indicate changes in reproductive effort in amphibians, where increased germinal epithelium depth correlates with increased effort by increasing the space for spermatogenesis to occur (Brannelly et al., 2021, 2016; McCallum and Trauth, 2007). During the testes and sperm staging procedures we were blinded to the experimental pathogen exposure status of the sample.

*Parasitic nematode collection*

The faecal smears were observed under a light microscope for the presence of adult female nematodes. If adult nematodes were found, they were counted and photographed. Because the frogs were fixed in formalin, only animals with adult nematodes were considered infected with intestinal nematodes. While nematode eggs can be identified using a faecal floatation test, this is not possible once samples have been fixed in formalin. However, because we analysed all the faeces within the intestine, we can be confident that if an adult female nematode was present, then we detected it.

*Morphological and molecular characterisation of nematodes*

The laboratory and bioinformatic pipeline carried out followed Huggins et al (2024b, 2024a), with the only adaptation being the use of the nematode-specific primers Nem_18S_R reverse complemented to be used as a forward primer (Floyd et al., 2005) and NC5 reverse complemented to be used as a reverse primer (Gasser et al., 1996). Primer sequences were forward primer: 5’– GCGATCAGATACCGCCC –3’ and reverse primer: 5’– AATGATCCTTCCGCAGGTTCACCTAC –3’ with the relevant Oxford Nanopore Technologies (ONT) adapter sequences added as per the manufacturer’s protocol. These primers generate an amplicon of ~750 bp.

*Statistical analysis*

All model results are presented in Appendix 2, Table S1. Below is a list of each model that was analysed based on the selection criteria presented in the main text.

*Disease dynamics.—* To assess *Bd* infection load over time we conducted a linear mixed effects model of *L. lesueuri* that had at least one positive weekly sample, where *Bd* infection load (log_10_ *Bd* DNA copies) is the response variable, week of the experiment, *Bd*-status (Infected/self-cleared from *Bd* infection), and individual was a random effect.

To assess the impacts of *Bd* on mortality of *L. lesueuri,* we conducted survival analyses using Cox regression to determine if either *Bd* infection status (infected through the experiment, cleared or uninfected) influenced survival over the 7-week (65 day) experiment, where survival (survived/died) before the end of the experimental period was the response variable, days survived (if the animal survived to the end of the experiment, the days survived was 65 days) and infection status (infected with *Bd*, cleared or never infected) were the predictor variables.

To assess whether *Bd* infection influenced the presence of intestinal nematodes, we conducted a Pearsons Chi-squared test where *Bd* infection status (the animal was infected with *Bd*, cleared from *Bd* infection for at least for at least one sample timepoint throughout the study or never returned a positive sample) was correlated with nematode presence at the end of the experiment (nematodes were detected / nematodes were not detected).

*Morphological assessment*.— We used a linear mixed effects model to assess the impact of infection on body condition, where size (mass, g) was the response variable, the fixed effects were week, *Bd* infection status and nematode presence and individual was a random effect. We used linear mixed effects models to assess the effect of *Bd* infection on arm width (mm) and thumb nuptial pad length (mm). The weekly arm width and thumb nuptial pad measurements were taken in triplicate and were analysed as an average for the individual’s weekly measurement. In these models, the response variables were arm size and thumb size. The fixed effects were week, *Bd* infection status, nematode presence and SVL. The random effect was individual frog.

*Testes morphology*.— A series of linear mixed effects models were conducted when there were multiple measurements/samples processed for an individual, where the response variables were area of the testis histosection, number of tubules within a histosection, average tubule area, maximum tubule area, and germinal epithelium depth. The fixed effects were *Bd*-status, nematode status, and the interactive effect of *Bd* infection status and nematode status*.* Individual was a random effect. Linear models were conducted when there was a single sample taken per individual, such as left testis length, where the testis was measured upon dissection.

*Sperm staging analysis*.— We used generalised linear mixed effects models with a beta distribution to analyse the relationship between *Bd* infection and the proportion of spermatogenesis cell clusters at the spermatogonia, spermatocyte, and spermatozoa stages. The fixed effects included infection status (infected, uninfected and cleared), parasite status (parasite negative and parasite positive) and frog mass, where the random effect was the individual frog. The overall proportion of spermatid cell clusters was low and in the field of view of some samples was equal to zero. Therefore, we did not assess spermatid proportions in these analyses.

***Results***

*Morphological identification of nematodes*

The female nematode specimens from this study collected from *L. lesueuri* are of similar size to *P. australiensis* and slightly larger than *P. lymnodynastes* (length 3.5 – 5.4 mm (mean 4.6 mm, n = 5) from *L. lesueuri*; 5.1 – 5.3mm (5.2 mm, n = 3) from *P. australiensis*; 3.2 – 4.3 mm (3.6 mm, n = 7) from *P. lymnodynastes*) but with overlapping ranges (published measurements from Inglis 1968a). Similarly, the mean oesophageal length of specimens from the current study (0.41 – 0.54 mm (0.47 mm, n = 5)) was greater than specimens of *P. australensis* (0.37 – 0.42 mm (0.39 mm, n = 3) and less than *P. lymnodynastes* 0.49 – 0.57 mm (0.52 mm, n = 7) but the ranges again overlap. There are slight distances in the length of the vulva from the anterior end (0.50 – 0.60 mm (0.53 mm, n = 5)) in *Parathelandros* sp. from this study, 0.43 – 0.49 mm (0.46 mm, n = 3) in *P. australiensis* and 0.62 – 0.83 mm (0.68 mm, n = 7) in *P. lymnodynastes*. The tail in specimens from this study (1.0 – 1.2 mm (mean = 1.1 mm, n = 5)) is similar in length to that of *P. australiensis* (1.19 – 1.29 mm (1.24 mm, n = 3)) but much longer than that of *P. lymnodynastes* (0.53 – 0.78 mm (0.62 mm, n = 7)). The specimens reported here are most similar to *P. australiensis*, based primarily on the length of the tail (Fig. S1).

*Molecular and phylogenetic characterisation of nematodes*

For the six samples that were analysed by nanopore sequencing, a total of 607,175 raw reads were obtained with a per sample mean (± standard error) of 101,196 (± 20,831). Following bioinformatic quality control, processing and clustering a total of 575,639 filtered reads were obtained across all samples with a per sample mean of 95,940 (± 19,013).

***References***

Berger, L., Marantelli, G., Skerratt, L.F., Speare, R., 2005. Virulence of the amphibian chytrid fungus *Batrachochytium dendrobatidis* varies with the strain. Dis Aquat Organ 68, 47–50.

Boyle, D.G., Boyle, D.B., Olsen, V., Morgan, J.A.T., Hyatt, A.D., 2004. Rapid quantitative detection of chytridiomycosis (*Batrachochytrium dendrobatidis*) in amphibian samples using real-time Taqman PCR assay. Dis Aquat Organ 60, 141–148.

Brannelly, L.A., Webb, R., Skerratt, L.F., Berger, L., 2016. Amphibians with infectious disease increase their reproductive effort: Evidence for the terminal investment hypothesis. Open Biol 6, 150251.

Brannelly, L.A., Webb, R.J., Jiang, Z., Berger, L., Skerratt, L.F., Grogan, L.F., 2021. Declining amphibians might be evolving increased reproductive effort in the face of devastating disease. Evolution 75, 2555–2567.

Brannelly, L.A., Wetzel, D.P., West, M., Richards-Zawacki, C.L., 2020. Optimized *Batrachochytrium dendrobatidis* DNA extraction of swab samples results in imperfect detection particularly when infection intensities are low. Dis Aquat Organ 139, 233–243.

Donnellan, S.C., Mahony, M.J., 2004. Allozyme, chromosomal and morphological variability in the *Litoria lesueuri* species group (Anura:Hylidae), including a description of a new species. Aust J Zool 52, 1–28.

Edgar, R.C., 2004. MUSCLE: Multiple sequence alignment with high accuracy and high throughput. Nucleic Acids Res 32, 1792–1797.

Floyd, R.M., Rogers, A.D., Lambshead, P.J.D., Smith, C.R., 2005. Nematode-specific PCR primers for the 18S small subunit rRNA gene. Mol Ecol Notes 5, 611–612.

Gasser, R.B., Stevenson, L.A., Chilton, N.B., Nansen, P., Bucknell, D.G., Beveridge, I., 1996. Species markers for equine strongyles detected in intergenic rDNA by PCR-RFLP, Molecular and Cellular Probes.

Hollanders, M., Royle, J.A., 2022. Know what you don’t know: Embracing state uncertainty in disease-structured multistate models. Methods Ecol Evol 13, 2827–2837.

Huggins, L.G., Atapattu, U., Young, N.D., Traub, R.J., Colella, V., 2024a. Development and validation of a long-read metabarcoding platform for the detection of filarial worm pathogens of animals and humans. BMC Microbiol 24.

Huggins, L.G., Colella, V., Young, N.D., Traub, R.J., 2024b. Metabarcoding using nanopore long-read sequencing for the unbiased characterization of apicomplexan haemoparasites. Mol Ecol Resour 24.

McCallum, M.L., Trauth, S.E., 2007. Physiological trade-offs between immunity and reproduction in the northern cricket frog (*Acris crepitans*). Herpetologica 63, 269–274.

R Core Team, 2022. R: A language and environment for statistical computing. R Foundation for Statistical Computing, Vienna, Austria. URL https://www.R-project.org/.

Ronquist, F., Teslenko, M., Van Der Mark, P., Ayres, D.L., Darling, A., Höhna, S., Larget, B., Liu, L., Suchard, M.A., Huelsenbeck, J.P., 2012. Mrbayes 3.2: Efficient bayesian phylogenetic inference and model choice across a large model space. Syst Biol 61, 539–542.

Rowley, J.J.L., Skerratt, L.F., Alford, R.A., Campbell, R., 2007. Retreat sites of rain forest stream frogs are not a reservoir for *Batrachochytrium dendrobatidis* in northern Queensland, Australia. Dis Aquat Organ 74, 7–12.

RStudio Team, 2020. RStudio: Integrated Development for R. RStudio, PBC, Boston, MA. URL http://www.rstudio.com.

Tamura, K., Stecher, G., Kumar, S., 2021. MEGA11: Molecular Evolutionary Genetics Analysis Version 11. Mol Biol Evol 38, 3022–3027.

West, M., Todd, C.R., Gillespie, G.R., McCarthy, M., 2020. Recruitment is key to understanding amphibian’s different population-level responses to chytrid fungus infection. Biol Conserv 241, 108247.

**Supplemental Figures**


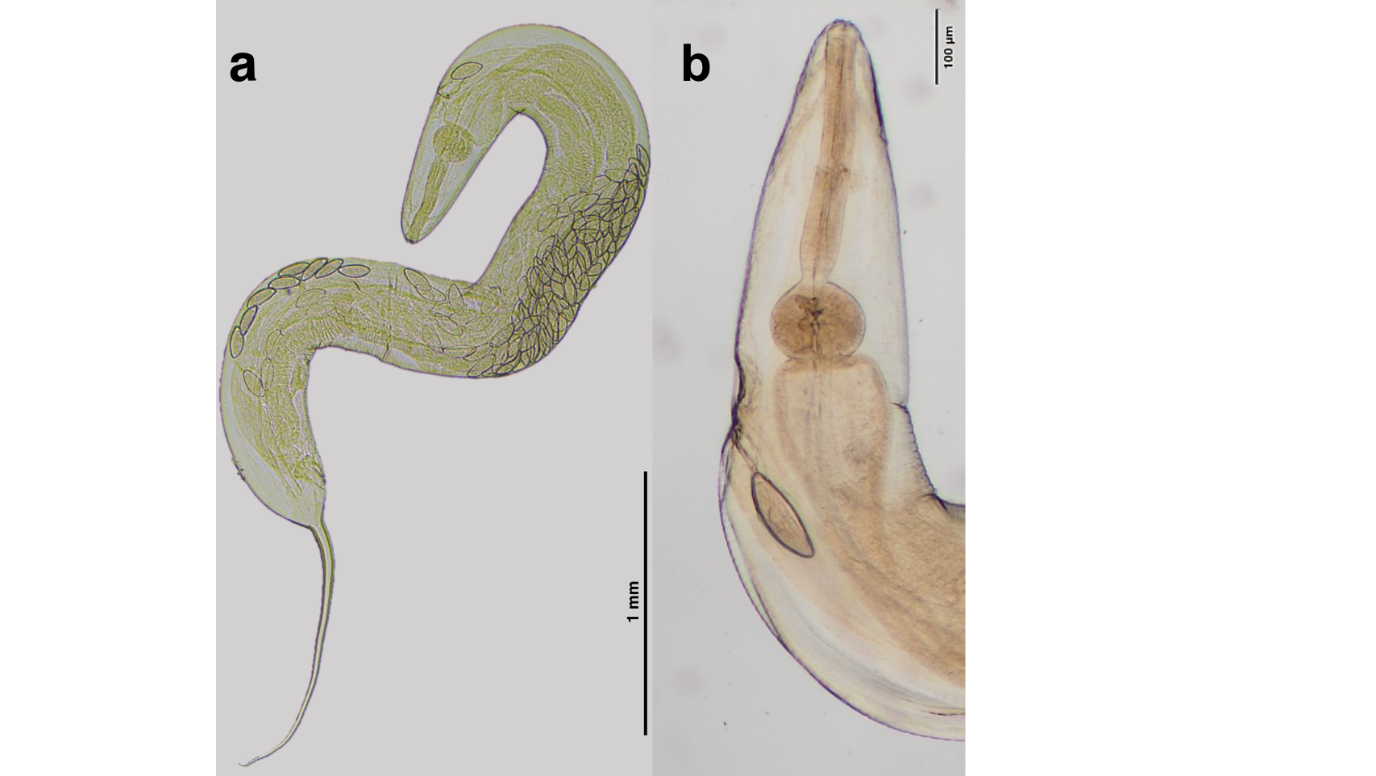


Figure S1. Photomicrographs of adult female *Parathelandro*s sp., lateral view. (a) Entire female. (b) Anterior extremity.
